# Behavioral profiling in children and adolescents with Malan syndrome

**DOI:** 10.3389/frcha.2023.1106228

**Published:** 2023-02-21

**Authors:** Paolo Alfieri, Federica Alice Maria Montanaro, Marina Macchiaiolo, Martina Collotta, Cristina Caciolo, Paolo Galassi, Filippo Maria Panfili, Fabiana Cortellessa, Marcella Zollino, Marcello Chinali, Maria Accadia, Marco Seri, Andrea Bartuli, Corrado Mammì, Marco Tartaglia, Stefano Vicari, Manuela Priolo

**Affiliations:** ^1^ Child and Adolescent Neuropsychiatry Unit, Department of Neuroscience, Bambino Gesù Children’s Hospital, IRCCS, Rome, Italy; ^2^ Rare Diseases and Medical Genetics Unit, University – Hospital Pediatric Department (DPUO), Bambino Gesù Children’s Hospital, IRCCS, Rome, Italy; ^3^ School of Pediatrics, University Tor Vergata, Rome, Italy; ^4^ Academic Department of Pediatrics, Bambino Gesù Children's Hospital, IRCCS, Rome, Italy; ^5^ Genetica Medica, Fondazione Policlinico Universitario A. Gemelli, IRCCS, Roma, Italy; ^6^ Dipartimento Universitario Scienze Della Vita e Sanità Pubblica, Sezione di Medicina Genomica, Università Cattolica del Sacro Cuore Facoltà di Medicina e Chirurgia, Roma, Italy; ^7^ Department of Pediatric Cardiology, Bambino Gesù Children's Hospital, Rome, Italy; ^8^ Medical Genetics Service, Hospital “Cardinale G. Panico”, Lecce, Italy; ^9^ Unit of Medical Genetics, IRCCS Azienda Ospedaliero Universitaria di Bologna, Bologna, Italy; ^10^ Operative Unit of Medical Genetics Bianchi – Melacrino- Morelli Hospital, Reggio Calabria, Italy; ^11^ Genetics and Rare Diseases Research Division, Bambino Gesù Children's Hospital, IRCCS, Rome, Italy; ^12^ Department of Life Sciences and Public Health, Università Cattolica Del Sacro Cuore, Rome, Italy

**Keywords:** malan syndrome, NFIX variants, neurobehavioral, rare genetic syndromes, intellectual disability, psychopathology, phenotype, genotype

## Abstract

Malan syndrome (MALNS) is an ultra-rare genetic disorder caused by heterozygous chromosomal microdeletions involving the 19p13.2 region or loss-of-function variants in the *NFIX* gene. It is characterized by specific phenotypical features, intellectual disability (ID), and limitations in adaptive functioning and behavioral problems. In a previous work, we defined the cognitive, adaptive, linguistic and visuomotor ability profiles in a group of 15 MALNS individuals, providing quantitative data from standardized evaluations. Here, we further extend the characterization of MALNS by analyzing the behavioral and psychopathological comorbidities of the same cohort, administering standardized tests. Children were evaluated from October 2020 to January 2022. Retrospective data analysis was also performed. Assessment consisted of clinical observations, structured parent interviews, and parent-reported questionnaires. For each scale, comparisons between subtests were performed. Results of our analysis show that the most prevalent psychiatric comorbidities are represented by anxiety symptoms (including GAD, separation anxiety and specific phobias), ADHD, autistic symptoms, and social and attention problems. Of note, minimal or no signs of ASD were observed. In conclusion, our findings indicate that the psychopathological and behavioral comorbidities, together with cognitive impairment, language problems and sensory difficulties interfere with development, daily activities and social participation, therefore contributing to the severity of the disability associated with MALNS. Awareness of this profile by professionals and caregivers can promote prompt diagnosis and support cognitive and behavioral development.

## 1. Introduction

Malan syndrome (MALNS) (MIM 614753) is an ultra-rare genetic disorder (1/1,000,000) caused by heterozygous chromosomal microdeletions involving the 19p13.2 region or loss-of-function (LoF) variants in the *NFIX* gene (nuclear factor I X) (OMIM 164005) (1). Variants and deletions generally occur as *de novo* events, although rare families segregating the disease have been reported due to either gonadal or parental mosaicism (2–4). NFIX belongs to the NFI family of DNA-binding proteins, which are highly conserved transcription factors controlling gene expression during brain and musculo-skeletal development (5–7). Individuals with *NFIX* microdeletions present with a significantly higher frequency of epilepsy and EEG anomalies than those carrying *NFIX* intragenic variants, possibly because of the involvement of adjacent genes contributing to the clinical phenotype in the frame of a contiguous gene disorder (2). No other clinically relevant genotype-phenotype correlation has been reported (8).

MALNS belongs to the family of overgrowth disorders, which are diseases characterized by excessive global or regional growth (9). More specifically, MALNS is characterized by postnatal overgrowth, occurring typically in childhood and adolescence, and involving preferentially the head circumference (macrocephaly/dolichocephaly observed >75% of individuals) and height (>2 SDs reported in >50% of patients). The final height during adulthood is less marked, falling within two SDs of the mean in two-thirds of individuals. Macrocephaly and peculiar dysmorphic features may raise clinical suspicion of MALNS, but genetic testing is required to confirm the clinical diagnosis.

MALNS individuals exhibit specific phenotypical features, mainly slender marfanoid habitus and skeletal abnormalities (i.e., pectus excavatum/carinatum, kyphoscoliosis, long and tapered fingers). Increased susceptibility to tibial fractures has also been observed. Facial features have been well characterized and are represented by a long and narrow face with a triangular shape, a high and prominent forehead, deeply set eyes, a depressed nasal bridge, a short nose with anteverted nares, everted lower lip with a small mouth and prognathia/prominent chin becoming more evident in adulthood (2, 8) Visual problems are frequent, especially strabismus, refractive errors, nystagmus, and optic nerve hypoplasia (8). Generalized hypotonia with swallowing difficulties may be present at birth (1, 2, 8). MALNS individuals may also present with brain abnormalities, such as enlarged ventricles, periventricular nodular heterotopias, hypoplasia of the corpus callosum, prominent cortical sulci, cavum septum pellucidum, cavum velum interpositi and Chiari I malformation (10, 11).

MALNS subjects typically show developmental delay (DD) and intellectual disability (ID), usually ranging from moderate to severe, though mild ID has been reported (2, 10–14). Likewise, adaptive functioning is usually lower than normal, generally ranging from moderately to severely impaired, with average communication skills the most affected. Indeed, linguistic skills are usually strongly damaged, even though receptive language seems to be more preserved than expressive one (15).

Behavioral abnormalities, such as anxiety, hyperactivity, hetero- and auto-aggressivity, and difficulties in coping with stress and novelty have also been described (2, 5, 10, 12). The onset of behavioral abnormalities is around ten years (5, 16), but no other studies about the onset age are available. Autistic traits, such as socio-relational difficulties and stereotypic behavior, have rarely been reported (2, 15).

Sensory processing difficulties, particularly hypersensitivity to visual and auditory stimuli, may also be present and contribute to the MALNS psychopathology, worsening anxious symptoms and challenging behaviors (2, 8, 14).

Cognitive impairment, together with behavioral problems and sensory difficulties, interfere with development, daily activities and social participation, therefore contributing to the adaptive functioning impairment and severity of the disability associated with MALNS, as established by the Diagnostic and Statistical Manual of Mental Disorders-fifth edition (DSM-5) (17).

It is well known that psychiatric disorders and behavioral abnormalities co-occur with ID, with several studies indicating a four- to five-fold increase in mental health problems (i.e., anxiety) among individuals with ID, particularly if severe (18).

Individuals with MALNS presenting with moderate to severe ID may be considered at risk from a psychopathological point of view. Indeed, psychiatric comorbidities enhance the severity of the presenting symptoms in MALNS, weighing on an adaptive functioning already compromised by physical and intellectual disability.

We previously outlined cognitive, adaptive, linguistic and visuomotor ability profiles in a cohort of 15 MALNS individuals (14). In the present retrospective study, we extend further the characterization of MALNS by defining the behavioral and psychopathological comorbidities. Specifically, we characterize the neurobehavioral phenotype of MALNS by providing standardized data, which are essential for comparisons between different studies and over time.

## 2. Materials and methods

### 2.1. Participants

Fifteen Italian subjects with a molecularly confirmed clinical diagnosis of MALNS (M/F = 9/6) were included in the study. Auxological and disease-related profiling of all subjects had previously been described (8), together with their cognitive, adaptive and linguistic features (14). Families involved in the study were middle-class (with some sporadic exceptions) with at least high-school education. All families were opposite gender parents and intact (conjugal or reconstituted), with just two children living in a single-parent household (mother).

The retrospective data were collected from records extracted from a dedicated database collecting pseudo-anonymized data that had been created for medical practice in the Child and Adolescent Psychiatry Unit of the Bambino Gesù Children's Hospital (Rome, Italy) and refer to a period between October 2020 and January 2022. Demographic data and IQ scores are summarized in Table 1.

**Table 1:** Gender, Age and IQ of participants. M, male; F, female; IQ, intelligence quotient.

*N*	Gender	Age	IQ
1	F	2.7	70^a^
2	M	6.4	65
3	F	7.3	54
4	M	7.11	58
5	M	8.1	52
6	F	8.7	54
7	M	8.11	40
8	M	11.3	62
9	M	13.3	42
10	M	13.10	69
11	F	15.5	50^b^
12	M	16.2	48
13	M	16.4	45
14	F	17.6	47
15	F	25.6	40

^a^ Developmental quotient.

^b^ Perceptual reasoning index.

### 2.2. Behavioral and psychopathological assessment

The assessment was conducted by a team of trained and specialized child psychiatrists, psychologists, and speech and language therapists, and consisted of clinical observations, standardized evaluations, parent interviews and parent-reported questionnaires. All the tests listed and described below were administered during routine clinical activities, usually lasting 3 working days.

#### 2.2.1. Schedule for affective disorders and schizophrenia for school-age children-present and lifetime version (K-SADS-PL-DSM-5)

K-SADS-PL-DSM-5 is a semi-structured interview for children between 6 and 17 years and for their parents that allows detecting current and life-time psychopathological/psychiatric symptoms according to DSM-5 criteria (19). In the case of patients with good language skills, K-SADS-PL was administered to both children and parents. However, due to the significant speech impairment of our sample, the interview was conducted with parents in most families, with children undergoing clinical observation only. For each subscale, items are scored “0” for no available information, “1” if the symptom is absent, “2” in case of sub-threshold symptom and “3” for threshold criterion.

#### 2.2.2. Children-Global assessment scale (C-GAS)

C-GAS (20) is a numeric scale ranging between 1 and 100 used by mental health clinicians to rate the general functioning of youths from 6 to 17 years of age. A clinically meaningful index is assigned to synthetize psychiatric symptomatology knowledge. Scores are grouped into ten categories used to summarise the level of functioning, ranging from “extremely impaired” (1–10) to “doing very well” (91–100).

Of note, C-GAS cannot be easily administered to individuals with ID and neurodevelopmental disorders, as those children follow abnormal developmental trajectories, often exhibiting severe impairments in specific areas of functioning. For this reason, we scored the scale only based on how much the psychopathological symptoms impact on functioning independently from the ID level.

#### 2.2.3. Children behavior check list (CBCL)

The behavioural profile was assessed using the CBCL scales (21) according to age: CBCL 1 ½–5 for one individual aged 2 years and 7 months and CBCL 6–18 for the others. CBCL generates eight syndrome scores: Anxious/Depressed, Withdrawn/Depressed, Somatic Problems, Social Problems, Thought Problems, Attention Problems, Rule Breaking, and Aggressive Behavior. Additionally, Competence Scale, Internalizing, Externalizing and Other Problems Scales, DSM-Oriented Scales and 2007 Scales scores, are calculated too.

According to the ASEBA Assessment Data Manager (ADM), *t*-scores of Syndrome Scales, DSM-Oriented Scale and 2007 Scales from 67 to 70 fall in the borderline range, while t-scores above 70 in the clinical range; concerning the Total Problem, Internalizing, and Externalizing Scale, t-scores of 60 to 63 delineate the borderline range, while t-scores above 63 delineate the clinical range.

#### 2.2.4. Conners parent rating scale-revised: long version (CPRS-R: L)

Child behavior was evaluated by using CPRS-R: L (22), which is a widespread research and clinical tool to obtain information about children's problems in the following seven subscales: Cognitive Problems (CP), Oppositional (O), Hyperactivity-Impulsivity (H-I), Anxious-Shy (A-S), Perfectionism (P), Social Problems (SP), and Psychosomatic (P). Additionally, different indexes, such as a global index, an Attention-Deficit and Hyperactivity Disorder (ADHD) index and a DSM–IV–TR related disorders index, are provided. CPRS-R: L is administered to children aged from 3 up to 17 years, while parents rated each item on a Likert scale from 0 (not true at all) to 3 (very much true). Raw scores are converted into T- scores, which have a mean (M) of 50 and a standard deviation (SD) of 10, and into percentile scores. Significant scores range from a low T-score of 61 (mildly atypical) to above 70 (markedly atypical).

#### 2.2.5. Childhood autism rating scale- second version (CARS-2)

CARS-2 (23) is a 15-item behavioural rating scale developed to measure the severity and to support the diagnosis of the autism spectrum disorder (ASD). The assessed items are: (1) Relating to People; (2) Imitation; (3) Emotional Response; (4) Body Use; (5) Object Use; (6) Adaptation to Change; (7) Visual Response; (8) Listening Response; (9) Taste, Smell, and Touch Response and Use; (10) Fear or Nervousness; (11) Verbal Communication; (12) Nonverbal Communication; (13) Activity Level; (14) Level and Consistency of Intellectual Response; and (15) General Impressions. Each item is scored from 1 (normal behaviour) to 4 (severely abnormal behaviour). A total raw score of 15 to 29.5 is considered non-autistic; a raw score of 30 to 36.5 is considered mild to moderate autism; a raw score from 37 to 60 is considered moderate to severe autism (24).

#### 2.2.6. Social communication questionnaire-lifetime version (SCQ-L)

SCQ-L (25) is a brief, 40-item, parent-report screening measure that focuses on items relating to ASD symptomatology likely to be observed by a caregiver. More specifically, the questionnaire allows us to detect if children over 4 years of chronological age or adults with a mental age of at least 2 years may exhibit ASD. Each item in the SCQ requires a dichotomous “yes”/“no” response. Each scored item receives a value of 1 point for abnormal behaviour and 0 points for the absence of abnormal behaviour/normal behaviour. SCQ-L does not aid in providing a diagnosis but is a useful tool to underline the necessity of a deeper evaluation. We used the recommended cut-off of 15 to indicate a possible ASD (26).

#### 2.2.7. Social responsiveness scale (SRS)

SRS is a widely used parent-report questionnaire that evaluates a child's social awareness, cognition, communication, motivation, and mannerisms (27). It comprehends 65 items scored from 0 (not confirmed) to 3 (almost always true) point Likert scale and can be used with children from 4 up to 18 years of age. SRS is not a diagnostic tool but can be used to support the diagnosis of ASD. Total raw scores are converted into T-scores to provide the relative normative position regarding social communication difficulties.

#### 2.2.8. Noise hypersensitivity

The presence or absence of hypersensitivity to auditory stimuli was investigated during the anamnestic, clinical interview, asking the patient's parents a targeted question.

## 3. Statistical analysis

Descriptive statistics (M, MD, min-max, SD) were calculated for age, CBCL 6–18, CPRS-R: L, CARS-2, SRS and SCQ scores. To explore any discrepancies within CBCL, CPRS-R: L and SRS sub-scales, the Wilcoxon Matched Pairs Test was used to test differences between pairs of subscales. Bonferroni's adjustment was applied to the Wilcoxon Matched Pairs Test to control multiple comparisons. All data analyses were performed using STATISTICA Six Sigma, STATISTICA release 7 (StatSoft, Inc., 1984–2006).

## 4. Results

### 4.1. Descriptive analysis

#### 4.1.1. K-SADS-PL-DSM-5 findings

K-SADS-PL-DSM-5 was administered to 14 out of 15 subjects in our cohort. One individual (subject 3) was excluded due to her age (2 years and 7 months). Our analysis revealed that anxiety spectrum symptoms were the most prevalent. More specifically, 5/14 subjects (36%) exhibited separation anxiety subthreshold symptomatology, plus one individual satisfying the threshold criterion for the disorder. Specific phobia traits were detected in 6/14 patients (43%), while Generalized Anxiety Disorder (GAD) traits were observed in 7/14 subjects (50%), plus another one meeting the criteria for the full disorder. Additionally, 4/14 subjects (29%) were diagnosed as affected with ADHD spectrum, and one exhibited ADHD traits only. Scores with lower prevalence were sub-threshold tics symptoms (2/14 individuals, 14%), enuresis (2/14, 14%), obsessive compulsive disorder (OCD) subthreshold criterion (1/14, 7%), traits of the Oppositional Deviant Disorder (ODD) (1/14, 7%), and encopresis (1/14, 7%). No psychopathological symptoms emerged in 2/14 individuals.

By combining the sub-threshold and supra-threshold criteria into one group, we observed that 5/14 subjects with MALNS had isolated psychopathological condition; 3/14 (21%) presented GAD only and 2/14 (14%) separation anxiety only. On the other hand, the most frequent comorbidities were GAD and specific phobias (5/14, 35%), separation anxiety and specific phobias (3/14, 21%), specific phobias and ADHD (3/14, 21%), GAD and ADHD (3/14, 21%).

#### 4.1.2. C-GAS findings

The same group evaluated with the K-SADS-PL-DSM-5 was also assessed through C-GAS. C-GAS mean (M) score was 57.7 (min-max 40–71; standard deviation, SD 11.7). C-GAS minimum score (40), index of serious problems, was found only in one patient, while the highest score in our sample (71) was assigned to 3/14 individuals (21%), indicating slight impairment.

Most of the subjects (6/14, 43%) felt in the range of scores from 41 to 50 (moderate degree of impairment), followed by three subjects (21%) in the range of 61–70 (some difficulties in a single area) and 1 in the range of 51–60, indicating variable functioning with sporadic difficulties.

#### 4.1.3. CBCL findings

Parents of 14 children aged 6 to 17 years completed the CBCL 6–18, while those of the youngest child out filled the CBCL 1 ½–5, therefore their answers were not included in our comparative analysis.

Descriptive statistics (M, median [MED], min-max, SD) about the 14 patients are reported in Table 2.

**Table 2:** Descriptive statistics of CBCL subscales. M, mean; MED, median; Min-Max, minimum-maximum; SD, standard deviation, **bold**: borderline range.

CBCL Subscales		M	MED	Min–Max	SD
***Competence scales***	Activities	30.7	27.5	20.0–64.0	11.5
	Social	33.4	28.5	20.0–52.0	10.9
	School	33.8	33.0	20.0–50.0	8.9
	Total Competence	26.1	24.0	13.0–48.0	10.8
***Syndrome scales***	Anxious/depressed	59.1	59.5	50.0–70.0	5.7
	Withdrawn/depressed	57.6	56.0	50.0–70.0	5.7
	Somatic complaints	61.5	61.0	53.0–82.0	8.5
	Social problems	**67**.**8**	69.0	53.0–84.0	8.1
	Thought problems	57.6	54.5	50.0–70.0	7.5
	Attention problems	62.4	61.5	52.0–77.0	7.6
	Rule-breaking behavior	54.5	53.0	50.0–62.0	4.3
	Aggressive behavior	55.3	54.5	50.0–70.0	6.0
***Internalizing, externalizing and total problems scales***	Internalizing	**62**.**0**	60.0	55.0–76.0	6.6
	Externalizing	52.3	54.0	40.0–68.0	8.3
	Total Problems	**60**.**6**	62.0	50.0–73.0	6.8
***DSM IV oriented scales***	Depressive problems	60.2	58.5	51.0–70.0	5.8
	Anxiety problems	**68**.**1**	70.0	51.0–74.0	6.3
	Somatic problems	58.4	55.5	50.0–87.0	10.6
	Attention deficit/hyperactivity	59.1	59.5	50.0–67.0	5.7
	Oppositional defiant	54.9	53.5	50.0–66.0	5.0
	Conduct problems	54.1	52.5	50.0–63.0	4.4
***2007 Scales***	Sluggish cognitive tempo	56.3	55.5	50.0–66.0	5.7
	Obsessive-compulsive problems	53.9	52.5	50.0–67.0	4.6
	Stress problems	59.7	61.0	50.0–74.0	7.6

Regarding Competence scales, the tested cohort obtained scores in the non-clinical range, with no differences between subscales. Concerning Syndrome scales, mean scores did not reach clinical significance. Social Problems were the only one in the borderline range (M = 67.8, SD = 8.1), with 5/14 subjects (35%) being at risk and 3/14 subjects (21%) showing clinical symptom scores. Additionally, some isolated clinical symptom scores were recorded on the Somatic complaints (2/14 patients) and Attention problems (2/14 patients) scales.

Concerning Internalizing, Externalizing and Total problems scales, only Internalizing problems and Total problems scales mean scores felt in the borderline range (respectively: M = 62.0 SD = 6.6, M = 60.6 SD = 6.8). In sum, 4/14 individuals (29%) had a score in a clinical range and were at risk, according to their Total Problems scores. For Internalizing Problems, 4/14 (29%) of the patients showed significant symptoms, 4/14 (29%) were at risk, with the remaining 6/14 (43%) showing scores in the non-clinical range. Concerning Externalizing Problems, only one subject out of 15 (7%) attained a clinically significant score, and 2/14 (14%) obtained a borderline score.

Additionally, any of the DSM IV-oriented scale's mean scores reached clinical significance. Only the Anxiety problems scale mean score was in the borderline range (M = 68.1, SD = 6.3), with 6/14 subjects (43%) scoring above the clinical cut-off and 4/14 (29%) falling in the at-risk range. Moreover, some isolated clinically significant scores were recorded on the Somatic Problems scale (2/14, 14%). Depressive problems scale registered only one patient (7%) over the clinical cut-off, plus 3/14 patients (21%) being at risk.

Lastly, the 2007 Scales mean scores did not reach clinical significance as well, with only one isolated clinical score on Stress Problems scale.

Concerning the individual assessed through the CBCL 1 ½–5, clinical symptom scores were observed on the Anxiety scale (T score 81), Developmental pervasive disorder scale (T score 81), Anxiety/depression scale (T score 79), Internalizing problems scale (T score 74), Emotional reactivity scale (T score 73) and Total Problems scale (T score 68).

#### 4.1.4. CPRS-R: L findings

CPRS-R: L 5 was filled by parents of 14/15 subjects in our cohort, as one individual was too young to be assessed with this scale. Table 3 depicts CPRS-R: L descriptive statistics (M, MED, min-max, SD).

**Table 3:** Descriptive statistics of CPRS-R:L subscales. M, mean; MED, median; Min–Max, minimum–maximum; SD, standard deviation, **bold**: borderline range, bold*: clinical range.

CPRS-R:L Subscales	M	MED	Min–Max	SD
A Oppositional	51.2	50.0	37.0–70.0	9.4
B Cognitive problems/Inattention	**72**.1*	70.0	52.0–99.0	14.0
C Hyperactivity	60.0	58.0	40.0–82.0	13.6
D Anxious-Shyness	**64**.**2**	67.5	39.0–90.0	15.0
E Perfection	51.9	48.0	39.0–75.0	10.5
F Social Problems	**63**.**8**	59.5	43.0–98.0	17.7
G Psychosomatic	52.0	53.0	40.0–63.0	7.5
H Conners’ADHD Index	**66**.**9**	66.5	51.0–95.0	12.8
I Conners’ Global Index Restless-Impulsive	58.3	57.0	42.0–77.0	10.6
J Conners’ Global Index, Emotional Lability	53.4	49.5	40.0–89.0	13.8
K Conners’ Global Index, Total	57.1	56.5	41.0–77.0	10.2
L DSM IV Symptom Subscales, Inattentive	**68**.**9**	68.0	50.0–96.0	12.7
M DSM IV Symptom Subscales, Hyperactive/Impulsive	59.5	57.0	39.0–87.0	14.5
N DSM IV Symptom Subscales, Total	**66**.**4**	68.5	47.0–96.0	14.1

The cognitive problems/inattention scale reached the clinical significance (M = 72.1, SD = 14), with 6/14 individuals (42%) above the conventional threshold and 5/14 (36%) in the mildly atypical level. Instead, Anxious-Shyness (M = 64.2, SD = 15), Social Problems (M = 63.8, SD = 17.7), Conners' Index (M = 66.9, SD = 12.8), DSM IV Symptom Subscale-Inattentive (M = 68.9, SD = 12.7) and DSM IV Symptom Subscale-Total (M = 66.4, SD = 14.1) approached conventional levels of significance.

Considering individual cases within the different subscales, 6/14 (42%) individuals exceeded the statistical significance in DSM IV Symptom Subscale (N scale). In comparison, 3/14 (21%) were assessed in the borderline level, indicating a high frequency of ADHD symptomatology. Interestingly, predominantly inattentive ADHD type (L scale) recorded clinically significant scores in 6 individuals out of 14 (43%) and borderline scores in 5 more subjects (36%), compared to only 3/14 (21%) subjects achieving conventional threshold levels in predominantly hyperactive/impulsive scale (M scale). Additionally, 5 out of 14 children (36%) were within the conventional bounds of statistical significance plus other five verging-on-significant in the Conners' ADHD Index (H scale).

Finally, our analysis revealed a high frequency of social problems (F scale) and hyperactivity (C scale), with respectively 5/14 (36%) and 4/14 (29%) above the edge of significance.

#### 4.1.5. CARS-2 findings

Our analysis showed that only 2/15 individuals (13%) exceeded the conventional cut-off of 29.5, indicating mild to moderate ASD symptomatology. The remaining 13 individuals (87%) showed minimal/no signs of ASD. Indeed, the mean CARS-2 total raw score was 25 (SD = 4.1, see Table 4), which indicates that average there were minimal or any signs of ASD symptoms.

**Table 4:** Descriptive statistics of SCQ, CARS-2, SRS and C-GAS. M, mean; MED, median; Min–Max, minimum–maximum; SD, standard deviation, **bold**: borderline range, bold*: clinical range.

Social communication, responsiveness and global functioning	M	MED	Min–Max	SD
SCQ total raw score	14.1	13.5	1.0–26.0	6.7
CARS-2 total raw score	25.0	25.5	19.0–34.0	4.1
SRS Social awareness domain (pT)	**64**.**5**	65.0	45.0–86.0	12.7
SRS Social cognition domain (pT)	**80**.8*	80.0	54.0–109.0	13.7
SRS Communication domain (pT)	**73**.2*	73.0	47.0–98.0	14.9
SRS Motivation domain (pT)	**70**.3*	67.0	39.0–101.0	17.2
SRS Autistic mannerisms domain (pT)	**84**.6*	83.0	59.0–112.0	15.0
SRS Total (pT)	**80**.3*	87.0	52.0–100.0	15.4
C-GAS score	57.7	54.5	40.0–71.0	11.7

Taking individual scores into consideration, Adaptation to Change, Fear or Nervousness and Verbal Communication subscales were the ones with the highest frequency of atypical behaviours, with 9/14 (64%) collecting a score greater than or equal to two. Just below, very common were Emotional response and body use scales, with respectively 8/14 (57%) and 7/14 (50%) subjects above the conventional cut-off. The remaining subscales scores were less recurrent.

#### 4.1.6. SCQ findings

Among 14 subjects (one was excluded due to age), five (36%) attained a score over the cut-off (15) to SCQ (M = 14.1, SD = 6.7).

#### 4.1.7. SRS findings

Our analysis revealed that all SRS scales mean T scores were above the significance cut-off (Table 4). More specifically, the most compromised SRS domain was the Autistic mannerisms domain (M = 84.6, SD = 15), followed by the Social cognition domain (M = 80.8, SD = 13.7), Communication domain (M = 73.2, SD = 14.9) and Social motivation domain (M = 70.3, SD = 17.2). The Social Awareness domain registered the lowest score (M = 64.5, SD = 12.7), indicating mild to moderate deficits.

#### 4.1.8. Noise hypersensitivity

Ten individuals out of 15 (67%) presented hypersensitivity to auditory stimuli according to an anamnestic interview.

### 4.2. Comparative analysis

Comparative analyses were performed within CBCL 6–18, CPRS-R: L and SRS sub-scales.

#### 4.2.1. Comparisons within CBCL subscales

**Figure 1:**
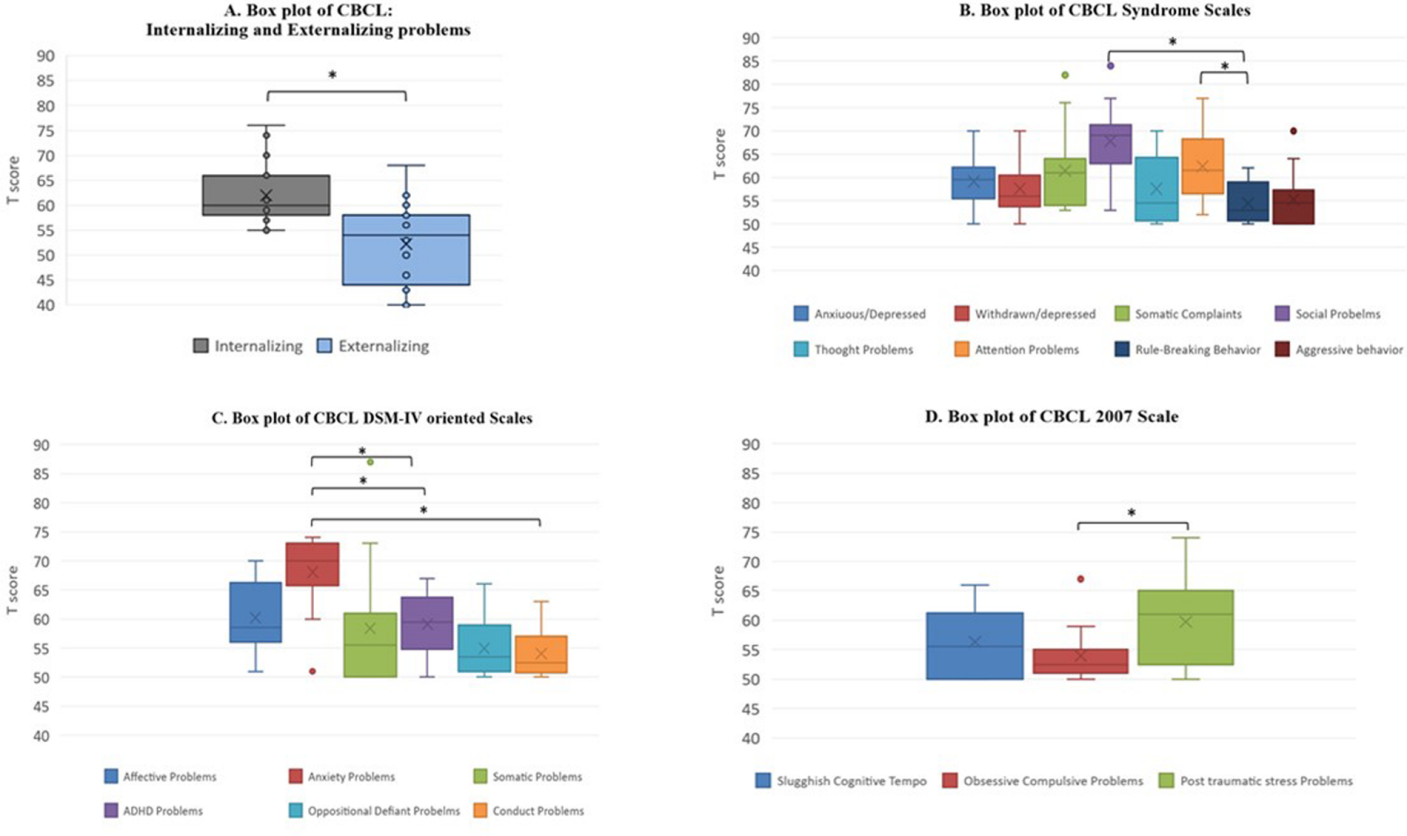
CBCL subscales comparisons (**A**). Internalizing, Externalizing and Total Problems scales (**B**). CBCL Syndrome Scales (**C**). DSM IV oriented scales (**D**). CBCL 2007 scale, *significant at *p* ≤ 0.05.

Comparisons were performed within the following: Competence Scales; Syndrome Scales; Internalizing, Externalizing and Total problems Scales; DSM-IV oriented Scales and 2007 Scales. Regarding Competence scales (Figure 1A), any of the subscales' comparisons reached statistical significance (*p* > 0.05 in all analyses). Instead, our analysis revealed significant results within two pairs of Syndrome Scales (Figure 1B). More specifically, the comparison between “Social Problems” and “Rule-breaking behaviour” eluded the conventional threshold of significance, with the first being dramatically greater. Additionally, to those results, another statistically meaningful difference was observed between “Attention problems” and “Rule-breaking behaviour”, with scores on the first being more clinically relevant than the ones on the second. In addition to those results, it emerged that the comparison between “Anxious/Depressed” and “Social Problems” barely failed to attain statistical significance, with higher mean scores in the latter than in the first. In the same way, scores on “Social Problems” resulted more elevated than the ones on “Aggressive behaviour”, even though the comparison was just reasonably close to significance. Furthermore, the difference between “Attention Problems” and “Aggressive behaviour” hovered on the brink of significance, too, with the first being clinically more impaired than the second.

Regarding Internalizing, Externalizing and Total problems scales, we observed that scores on the “Internalizing problems” subscale were significantly more significant than the ones on the “Externalizing problems” one (Figure 1A). Moreover, considering DSM IV oriented scales, we observed other significant differences. In particular, “Anxiety problems” were clinically more meaningful than “Attention-deficit/hyperactivity”, than “Oppositional deviant”, and then “Conduct problems”. Besides, a positive trend toward significance was observed by the comparison between the “Affective problems” and “Conduct problems” subscales, with scores on the latter being averagely less clinically crucial than the ones on the first (Figure 1C).

Finally, by examining the 2007 scales, it emerged that the “Stress Problems” subscale collected mean scores significantly higher than the ones on the “Obsessive-compulsive problems” subscale (Figure 1D).

#### 4.2.2. Comparisons within SRS subscales

**Figure 2:**
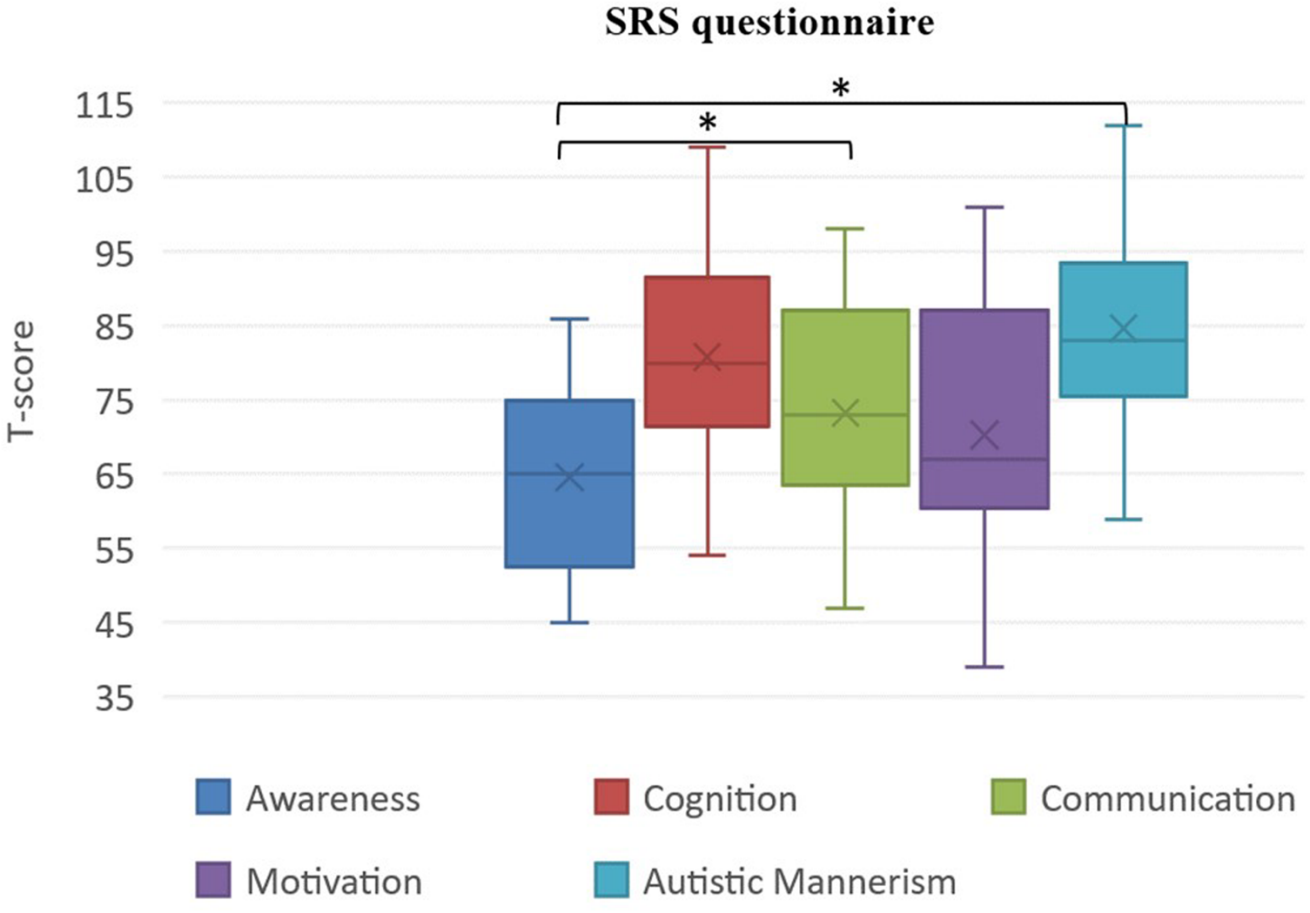
SRS subscales comparisons. *Indicates statistical significance of comparisons (*p* ≤ 0.05).

As Figure 2 shows, significant differences emerged between “Awareness” and “Communication”, with the second being dramatically more impaired than the first. The “Awareness” domain was significantly lower also than the “Autistic mannerism” one. No other significant differences emerged from our analysis.

#### 4.2.3. Comparisons within CPRS-R:L subscales

Comparative analysis within CPRS-R: L subscales did not show a clinically significant difference (*p* > 0.05 in all analyses).

## 5. Discussion

Pediatric and adult individuals with physical and intellectual disabilities are known to be more at risk for psychiatric comorbidities than the general population (28). On the other side, people with ID not infrequently receive an unidentified psychiatric diagnosis, as clinicians often attribute the symptoms of the psychiatric disorders to ID, underestimating the chance that their symptoms might be related to specific psychopathology (29).

In MALNS, ID is invariably present, together with other language deficits and additional physical disabilities, urging an early evaluation of behavior and psychopathological traits in each affected individual to characterize every single cognitive-behavioral phenotype better and to address proper treatment. We strongly believe that a deep characterization of the psychological aspects of MALNS is essential for a better comprehension of this condition. Psychopathological assessment and diagnosis of MALNS individuals may be challenging because of the interplay among ID, physical disability, language impairment and sensory abnormalities in conditioning the neurobehavioral profile. In turn, anxious symptoms, attentional difficulties and socio-relational impairment may interact with each other and negatively affect social behavior. This clinical complexity must be considered to avoid misleading diagnostic labels, i.e., language impairment and social anxiety may represent confounders when evaluating ASD in *x* individuals.

The main aim of the present study was to outline the behavioral and psychopathological comorbidities of a cohort of 15 MALNS subjects, whose cognitive profile had previously been characterized (14). We used standardized tests to allow comparisons between different studies on various ID syndromic conditions and, eventually, to be used in longitudinal studies.

K-SADS evaluation shows that the most prevalent psychiatric comorbidities are represented by anxiety symptoms (including GAD, separation anxiety and specific phobias), ADHD and autistic symptoms. Anxiety disorders don't seem confined to a particular range of age, representing a frequently reported comorbidity from early childhood to adolescence. In contrast, autistic symptoms and ADHD represent the second most frequent psychopathology of preschool and school age, respectively. Other disorders with lower prevalence have been detected too, which means that a complete psychopathological investigation is essential due to the heterogeneity of the psychopathological aspects of MALNS. Interestingly, the average C-GAS range score was 41–50, indicating that psychopathological symptoms moderately interfere with personal functioning, sharply contrasting with ID. Indeed, in support of the cognitive characterization offered by our previous work (14), the CPRS-R:L scale revealed that only the cognitive problems/inattention subscale reached the clinical significance, confirming that the low cognitive profile is a main and highly impacting feature of MALNS.

Considering CARS-2 results, we observed minimal or no signs or ASD in our cohort. The Verbal Communication subscale was particularly impaired, confirming the expressive language difficulties that were previously deeply described (14). We speculate that verbal communication difficulties impact the social interaction of MALNS people. We evidence that, despite their motivation for interpersonal relationships, they eventually lack effective communication, manifesting socio-relational atypia such as in ASD, without fulfilling ASD criteria [DSM-5] (17). This data is also in line with the analysis of the SRS, where the “social awareness” and “social motivation” domains were relatively spared compared to the “communication” and the “autistic mannerism” domains, suggesting a preserved motivation for interpersonal relationships even when language problems or stereotyped movements were reported.

Furthermore, by examining the CBCL subscales comparisons, we found that “Social problems” and “Attention Problems” scores were significantly greater than ones on “Rule-breaking behaviour”, pointing once again that cognitive and social impairment have a high burden in MALNS people. Additionally, taking into consideration the comparisons that barely failed to attain statistical significance, we observed how “social” and “attention problems” subscales received higher scores than “aggressive behavior”, in accordance with what emerged by the analysis of “internalizing, externalizing and total problems scales”, in which internalizing symptoms resulted significantly higher concerning the externalizing ones. This data also agrees with DSM IV-oriented scales comparisons analysis showing that “Anxiety problems” scores were dramatically higher than “Oppositional deviant” and “Conduct problems” ones. Altogether, these results may be interpreted as follows: Social and cognitive impairment, as well as anxiety, seem central and distinctive features in MALNS behavioural profile; besides, ID, together with language impairment may bring MALNS individuals to be unable to express emotions, therefore preferring the social withdrawal and shyness rather than to communicating psychological and emotional issues and exhibiting autistic behaviours without satisfying the criteria for ASD diagnosis.

Another intriguing data is similar results in K-SADS, CPRS-R: L and CBCL regarding ADHD, particularly in its predominantly inattentive form, representing the second most frequently reported comorbidity, just after anxiety symptoms. This result is significant, as ID, anxiety, language impairment, and sensory difficulties may be more prominent, with the risk of underestimating ADHD diagnosis. This observation should be considered both during clinical assessment and in the educational/rehabilitative context, as attention deficits may impair school learning and the reaching of personal autonomies.

Summing up, the collected data evidence that the psychopathological profile of MALNS is mainly characterized by anxiety and ADHD and that the interplay between ID, verbal communication deficits and sensory abnormalities, particularly hypersensitivity to auditory stimuli, may lead to MALNS individuals to enact autistic behaviors, to social withdraw and to shut themselves off.

While acknowledging the clinical complexity of MALNS neurobehavioral phenotype, we believe that an adequate understanding of the psychopathology associated with MALNS will allow a more comprehensive clinical approach, oriented to symptom identification, detection of comorbidities, and administration of more effective treatments and earlier interventions, eventually leading to better clinical outcomes. Cognitive-behavioral therapy (CBT) has been proven effective in treating anxiety disorders (30), ASD (31) and ADHD (32), therefore the early start of CBT mostly in case of patients with adequate cognitive skills, could be recommended. Indeed, it appears to be a well-tolerated treatment in people with mild ID with maladaptive and interfering anxiety (33, 34). Another valid intervention opportunity is augmentative alternative communication (AAC), whose appliance could accommodate the communicative intentionality of MS patients hidden by language difficulties, facilitating their interpersonal relationships.

Finally, any drug therapy to treat psychiatric comorbidities should carefully take into consideration the peculiarities of MALNS subjects, either in terms of age (childhood and adolescence) and the possible interactions with other drugs eventually administered to these subjects for their additional medical conditions.

To the best of our knowledge, this is the first systematical study aiming to define the behavioral and psychopathological profile of MALNS individuals, according to standardized tests and structured clinical interviews, therefore representing a point of strength of our work. Due to the moderate to severe intellectual disability and the language impairment of the individuals evaluated, patient-reported data are lacking. Moreover, no standardized psychopathology scale is tailored explicitly on intellectually disabled people. We, therefore, collected data from both clinical-reported and parent-reported interviews, which turned out to be concordant in describing the psychopathological traits and their impact on the patient's global functioning. However, the absence of systematized method to collect socio-economic status of families involved represents a limitation of our study. Moreover, because of the small size of the sample, a wider series evaluation with systematical data collection and longitudinal follow-up, may represent interesting study perspectives.

## 6. Conclusion

In subjects with MALNS, behavioral and psychopathological comorbidities (i.e., anxiety and ADHD symptoms) aggravate the disability and impairment of adaptive functioning. Providing the referring clinician with a neurobehavioral profile of the patient may help to depict the most prevalent behavioral and psychopathological anomalies to facilitate a prompt diagnosis and more effective management providing a tailored and early intervention. Reducing the impact of psychiatric comorbidities would ensure a better quality of life for these patients and their families. In the light of this new evidence, we suggest that clinical practice should prioritize the evaluation of developmental/cognitive/adaptive profiles and of psychopathological symptoms/traits, when assessing people with MALNS (see Supplementary Table).

In conclusion, treatment of MALNS should be multidisciplinary, aimed at managing physical, cognitive and psychological problems, to improve the outcome of affected individuals.

## Data Availability

The original contributions presented in the study are included in the article/**Supplementary Material**, further inquiries can be directed to the corresponding author.
