# Supplementary material for: Behavioral profiling in children and adolescents with Malan syndrome
Source: Front Child Adolesc Psychiatry. 2023 Feb 21;2:1106228. doi: 10.3389/frcha.2023.1106228 (PMC11732151; doi:10.3389/frcha.2023.1106228)
Supplement: Supplementary file 1 [file Table2.docx]

|  | **Toodler**  (12-36 months) | **Preschooler**  (3.1-6 years) | **School age child**  (6.1-10 years) | **Adolescent**  (10.1-19 years) | **Adult**  (above 19.1) | **Suggestion for follow-up**  (timing) |
| --- | --- | --- | --- | --- | --- | --- |
| **Psychomotor/**  **cognitive abilities** | *Developmental assessment* (i.e. Griffiths Scales of Child Development*; Bayley Scales of Infant and Toddler Development*) | *Cognitive assessment*  (i.e. Leiter International Performance Scale* for non verbal children; Kaufman Assessment Battery for children* or Wechsler Preschool and Primary Scale of Intelligence* for verbal children) | *Cognitive assessment*  (i.e. Leiter International Performance Scale* for non verbal children; Kaufman Assessment Battery for children* or Wechsler Intelligence Scale for Children* for verbal children) | *Cognitive assessment*  (i.e. Leiter International Performance Scale* for non verbal adolescent; Kaufman Assessment Battery for children* or Wechsler Intelligence Scale for Children* below 17 years; Wechsler Adult Intelligence Scale* above 16.11 years for verbal adolescent) | *Cognitive assessment*  (i.e. Leiter International Performance Scale* for non verbal adult; Wechsler Adult Intelligence Scale* for verbal adult) | From 12 to 36 months: follow-up approximatively every 1 years;  From 3.1 to 6 years: follow-up approximatively every 2 years |
| **Adaptive behavior** | *Adaptive assessment*  (i.e. Vineland Adaptive Behavior Scale*; Adaptive Behavior Assessment System*) | *Adaptive assessment*  (i.e. Vineland Adaptive Behavior Scale*; Adaptive Behavior Assessment System*) | *Adaptive assessment*  (i.e. Vineland Adaptive Behavior Scale*; Adaptive Behavior Assessment System*) | *Adaptive assessment*  (i.e. Vineland Adaptive Behavior Scale*; Adaptive Behavior Assessment System*) | *Adaptive assessment*  (i.e. Vineland Adaptive Behavior Scale*; Adaptive Behavior Assessment System*) | From 12 to approximatively 36 months: follow-up approximatively every 1 years;  From 3.1 to 6 years: follow-up approximatively every 2 years |
| **Communication/**  **Language abilities** | *Parent report questionnaire*  (i.e. MacArthur-Bates. Communicative Development Inventory)°  *Structured evaluation*  -Language comprehension°  -Language production°  -Evaluation of speech and sound disorders (articulation and phonology)° | *Structured evaluation*  -Language comprehension°  -Language production°  -Evaluation of speech and sound disorders (articulation and phonology)° | *Structured evaluation*  -Language comprehension°  -Language production°  -Evaluation of speech and sound disorders (articulation and phonology)° | *Structured evaluation*  -Language comprehension°  -Language production°  -Evaluation of speech and sound disorders (articulation and phonology)° | *Structured evaluation*  -Language comprehension°  -Language production°  -Evaluation of speech and sound disorders (articulation and phonology)° | From 12 to approximatively 36 months: follow-up approximatively every 1 years;  From 3.1 to 6 years: follow-up approximatively every 2 years |
| **Psychopathological symptoms/traits of neurodevelopmental disorders (i.e. ASD, ADHD etc.)** | *Parent report questionnaire*  (i.e. Child Behavior Checklist for Ages 1 ½-5)  *Structured evaluation*  (i.e. Autism Diagnostic Observation Schedule second edition, above 1 year or  Childhood Autism Rating Scale, second edition, above 2 years) | *Parent report questionnaire*  (i.e. Child Behavior Checklist for Ages 1 ½-5)  *Structured evaluation*  (i.e. Autism Diagnostic Observation Schedule second edition, above 1 year or  Childhood Autism Rating Scale, second edition, above 2 years, SRS - Social Responsiveness Scale  Above 4 years) | *Parent report questionnaire*  (i.e. Child Behavior Checklist for Ages 6-18)  *Structured evaluation*  (i.e. Autism Diagnostic Observation Schedule second edition, above 1 year or  Childhood Autism Rating Scale, second edition, above 2 years, SRS - Social Responsiveness Scale  Above 4 years) | *Parent report questionnaire*  (i.e. Child Behavior Checklist for Ages 6-18)  *Structured clinical interview*  [(i.e. Kiddie Schedule for Affective Disorders and Schizophrenia* + Children-Global Assessment Scale)](https://en.wikipedia.org/wiki/Kiddie_Schedule_for_Affective_Disorders_and_Schizophrenia)  *Structured evaluation*  (i.e. ADOS-2 – Autism Diagnostic Observation Schedule second edition, above 1 year or  Childhood Autism Rating Scale, second edition, above 2 years, SRS - Social Responsiveness Scale  Above 4 years) | *Structured clinical interview*  (i.e. Kiddie Schedule for Affective Disorders and Schizophrenia* + Children-Global Assessment Scale)  *Structured evaluation*  (i.e. Autism Diagnostic Observation Schedule second edition, above 1 year or  Childhood Autism Rating Scale, second edition, above 2 years) | From 12 to approximatively 36 months: follow-up approximatively every 1 years;  From 3.1 to 6 years: follow-up approximatively every 2 years |

*latest edition available according to the country in which the evaluation takes place

°tests and normative data available according to the country in which the evaluation takes place
